# Nitric Oxide Enhances Desiccation Tolerance of Recalcitrant *Antiaris toxicaria* Seeds via Protein S-Nitrosylation and Carbonylation

**DOI:** 10.1371/journal.pone.0020714

**Published:** 2011-06-02

**Authors:** Xuegui Bai, Liming Yang, Meihua Tian, Jinhui Chen, Jisen Shi, Yongping Yang, Xiangyang Hu

**Affiliations:** 1 Key Laboratory of Biodiversity and Biogeography, Kunming Institute of Botany, Institute of Tibet Plateau Research at Kunming, Chinese Academy of Science, Kunming, China; 2 School of life science, Huaiyin Normal University, Huai'an, China; 3 Key Laboratory of Forest Genetics & Biotechnology, Nanjing Forestry University, Ministry of Education, Nanjing, China; University of Melbourne, Australia

## Abstract

The viability of recalcitrant seeds is lost following stress from either drying or freezing. Reactive oxygen species (ROS) resulting from uncontrolled metabolic activity are likely responsible for seed sensitivity to drying. Nitric oxide (NO) and the ascorbate-glutathione cycle can be used for the detoxification of ROS, but their roles in the seed response to desiccation remain poorly understood. Here, we report that desiccation induces rapid accumulation of H_2_O_2_, which blocks recalcitrant *Antiaris toxicaria* seed germination; however, pretreatment with NO increases the activity of antioxidant ascorbate-glutathione pathway enzymes and metabolites, diminishes H_2_O_2_ production and assuages the inhibitory effects of desiccation on seed germination. Desiccation increases the protein carbonylation levels and reduces protein S-nitrosylation of these antioxidant enzymes; these effects can be reversed with NO treatment. Antioxidant protein S-nitrosylation levels can be further increased by the application of S-nitrosoglutathione reductase inhibitors, which further enhances NO-induced seed germination rates after desiccation and reduces desiccation-induced H_2_O_2_ accumulation. These findings suggest that NO reinforces recalcitrant seed desiccation tolerance by regulating antioxidant enzyme activities to stabilize H_2_O_2_ accumulation at an appropriate concentration. During this process, protein carbonylation and S-nitrosylation patterns are used as a specific molecular switch to control antioxidant enzyme activities.

## Introduction

Recalcitrant seeds, also known as unorthodox seeds, lose viability following exposure to either drying or freezing conditions after being shed from the parent plant. Unlike orthodox seeds, recalcitrant seeds are not well suited to long-term storage, for example, in germplasm repositories [1], [2]. Therefore, the nature of recalcitrant seeds presents practical challenges for seed maintenance and genetic conservation. To date, our understanding of the mechanisms underlying the intolerance of recalcitrant seeds to either drying or freezing has been limited. Recent evidence has indicated that reactive oxygen species (ROS), particularly H_2_O_2_, derived from aberrant metabolic activity, damage intracellular structures in recalcitrant seeds [3], [4], [5]. The glutathione-ascorbate cycle is a metabolic pathway that can efficiently detoxify H_2_O_2_. This pathway involves antioxidant metabolites including ascorbate, glutathione and NADPH, and the enzymes linking these metabolites pathway include ascorbate peroxide (APX), glutathione reductase (GR), monodehydroascorbate reductase (MDAR) and dehydroascorbate reductase (DHAR). In the first step of this pathway, H_2_O_2_ is reduced to water by APX using ascorbate as the electron donor. The oxidized ascorbate (monodehydroascorbate or dehydroascorbate) is regenerated by MDAR and DHAR at the expense of reduced glutathione (GSH), yielding oxidized glutathione (GSSG). Finally, GSSG is reduced by GR, using NADPH as the electron donor. The reduction of dehydroascorbate may be non-enzymatic or catalyzed by proteins with DHAR activity, such as glutathione-S-transferase (GST) or glutaredoxins [6]. In plants, glutathione, ascorbate and NADPH are present in high concentrations; it is assumed that the glutathione-ascorbate cycle plays a key role in H_2_O_2_ detoxification [4].

Nitric oxide (NO) is a gaseous free radical and signaling molecule that participates in multiple aspects of plant development, including the vegetative to floral transition, root growth and gravitropism, adventitious root formation, xylogenesis, pollen tube growth and stomatal closure [3], [7], [8], [9], [10], [11]. The animal nitric oxide synthase (NOS)-like enzyme and nitrate reductase (NR) are thought to be responsible for NO generation in plants, although the genes encoding plant NOS-like enzymes remain elusive. Apoplastic synthesis of nitric oxide has also been suggested as a source of NO [12]. Current evidence indicates that NO closely interacts with many signaling molecules usually involved in plant adaptive stress responses, including ABA (abscisic acid) and ROS. For example, ABA-induced NO generation and stomatal closure in Arabidopsis are dependent on H_2_O_2_ synthesis [13]. NO also significantly improves the plant antioxidative capacity against ROS damage [14], [15], [16]. There is growing evidence that, as in animals, the S-nitrosylation of plant proteins is important to regulation of a wide range of cellular events [17]. Recently, Tanou *et al.* [18], [19] reported that salt-induced protein carbonylation, a type of protein oxidation that can be promoted by ROS, could be alleviated by NO pretreatment, suggesting that protein carbonylation and S-nitrosylation patterns are specific molecular indicators of plant vigor under stressful conditions[18], [19]. These findings imply that protein modifications by carbonylation or S-nitrosylation at the post-transcriptional level are novel ways for plants to respond to environmental stress.

*Antiaris toxicaria* Lesch is a monoecious tree that grows to 25–40 m in height with a trunk up to 40 cm in diameter, and it is best known as the source of cardenolide arrow poison, as alkaloids in its sap cause cardiac arrest. Its fruits are either orange or dark red indehiscent drupes covered with velvety hairs. The seeds are broad, approximately 10 mm long, and are recalcitrant, showing sensitivity to either desiccation or chilling during germination [20], [21], [22]. NO has been previously reported to promote seed germination and reduce seed dormancy in *Arabidopsis* and grass seeds [20], [21], [23], [24]. Here, we found that NO efficiently abolishes desiccation-induced inhibitory effects on *Antiaris toxicaria* seed germination. NO also stimulates the activity of antioxidant enzymes involved in the ascorbate-glutathione metabolism cycle, and proteomics analysis revealed that the NO-mediated balance of protein carbonylation and S-nitrosylation status plays an essential role in the regulation of antioxidant enzymes and the stabilization of the ROS concentration at an acceptable level. Our results reveal the novel role of protein carbonylation and S-nitrosylation in seed physiology and suggest that NO is a potential new tool for recalcitrant seed conservation.

## Results

### NO reduced H_2_O_2_ accumulation in *A.toxicaria* seeds responding to desiccation

**Figure 1:**
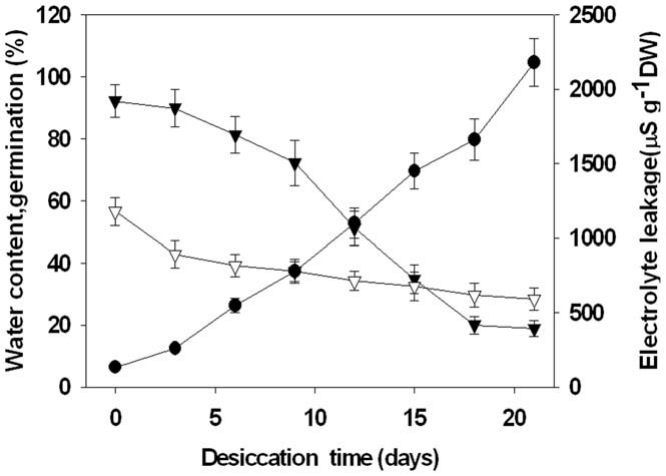
Changes in water content (▽), germination percentage (▾) and electrolyte leakage (○) during the drying of *Antiaris toxicaria* seeds.

**Figure 2:**
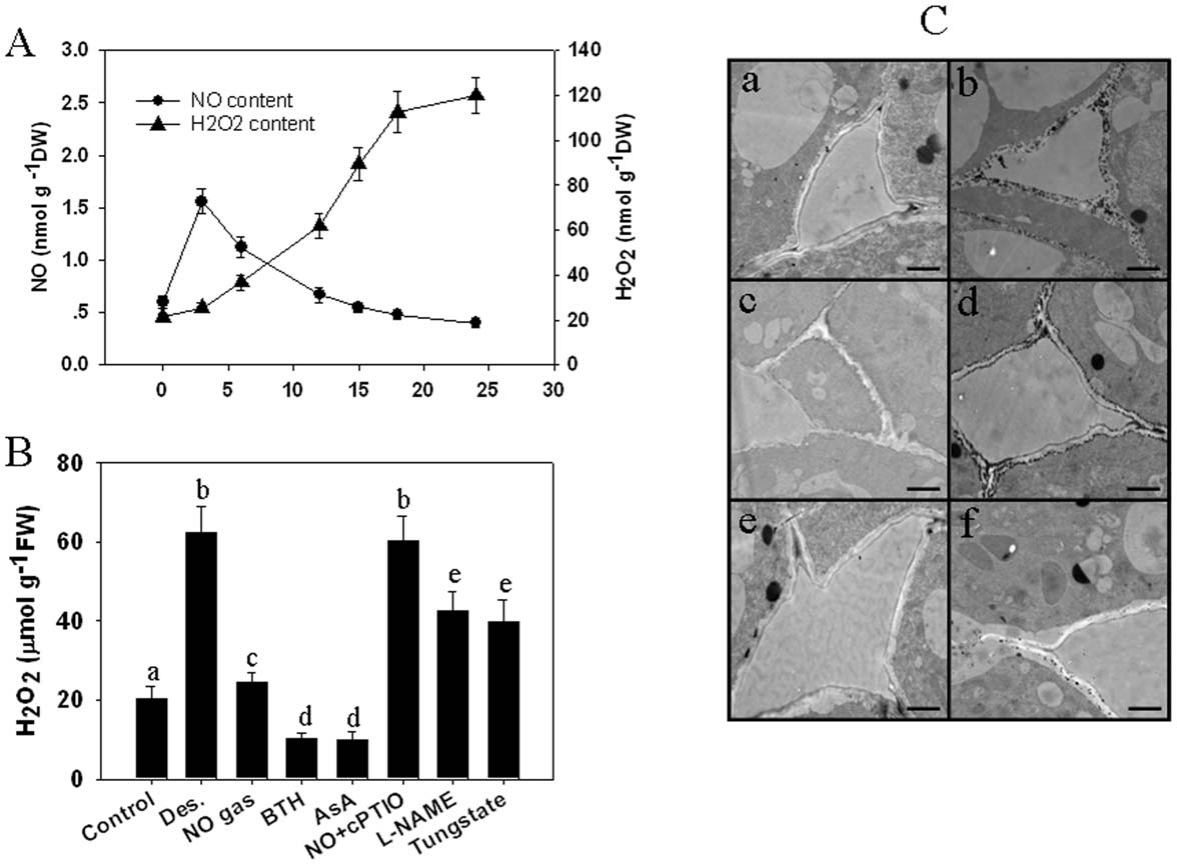
Desiccation induced the generation of NO and H_2_O_2_ in the embryos of *A. toxicaria* seeds. **Data means ±SE (n = 30), are representative of explants from at least three independent experiments.** **A**) Time-course of NO and H_2_O_2_ accumulations in the embryos of *Antiaris toxicaria* seeds during desiccation. **B**) Effects of NO gas, ROS and NO scavengers, and NO metabolism inhibitors on desiccation-induced H_2_O_2_ accumulation in embryos of *Antiaris toxicaria* seeds. The shedding seeds from *Antiaris toxicaria* were collected immediately and treated with different concentrations of chemicals (1 mM AsA; 1 mM BTH; 10 µM cPTIO; 20 µM L-NAME; 50 µM tungstate) for 12 h prior to 12 days of desiccation or NO gas exposure. After desiccation, the seed embryos were immediately isolated, and the H_2_O_2_ content was measured. Data analyzed by one-way ANOVA followed by Tukey's test, different symbols indicate significant differences between treatments (*P*<0.05). **C**) Subcellular localization of H_2_O_2_ accumulation in seed embryos by CeCl_3_-based staining with transmission electron microscopy. a: control, freshly shed seed embryo; b: seed embryo after 12 days of desiccation; c: seed embryo after 12 days of desiccation plus 100 ppm NO gas exposure; d: seed embryo pretreated with 10 µM cPTIO for 12 h followed by 12 days of desiccation plus 100 ppm NO gas exposure; e: seed embryo pretreated with 1 mM AsA for 12 h followed by 12 days of desiccation; f: seed embryo pretreated with 1 mM BTH for 12 h followed by 12 days of desiccation.

During the experiment, the relationship among water loss, seed germination rate and electrolyte leakage was investigated in *A. toxicaria* recalcitrant seeds after air-drying. As shown in Figure 1, freshly shed seeds exhibited a high water content (56.2±2.1%) and germination rate (92.4±5.1%). During the course of desiccation by air-drying, the germination capability declined with the loss of water, and the decline was accompanied by increasing electrolyte leakage, which result in membrane disintegration. The increase in H_2_O_2_ generation was clear and sustained during desiccation (Figure 2A); NO release also showed a rapid increase at the beginning of water loss, but its release decreased after three days of desiccation treatment (Figure 2A). A strong correlation was found among seed viability determinants such as seed germination and H_2_O_2_ and NO levels after three days of desiccation (Table S1), which indicates that both H_2_O_2_ and NO are involved in the mechanism of seed intolerance to desiccation. To further explore this idea, we first investigated the effect of NO on desiccation-induced H_2_O_2_ production in *A. toxicaria* embryos. As shown in Figure 2B, desiccation quickly induced the accumulation of H_2_O_2_, which could be repressed by exposure to NO gas. Removal of NO by the NO scavenger 2-(4-carboxyphenyl)-4,4,5,5–tetram-ethylimidazoline-1-oxyl-3-oxide (cPTIO) could reverse the inhibitory effect of NO on H_2_O_2_ accumulation. Retreating the seeds with reduced AsA (ascorbate acid) also reduced desiccation-induced H_2_O_2_ accumulation. NG-nitro-l-arginine methyl ester (L-NAME) and tungstate are specific inhibitors of NOS-like and NR enzymes. We found that pretreatment with L-NAME and tungstate also repressed desiccation-induced H_2_O_2_ generation (Figure 2B). Subcellular localization of H_2_O_2_ accumulation in desiccated seeds was detected by a CeCl_3_-based cytochemical technique in which the CeCl_3_ reacts with H_2_O_2_ to form electron-dense deposits of cerium perhydroxide that are detected by electron microscopy. We found very few deposits of cerium perhydroxide prior to desiccation (Figure 2C upper left), whereas considerable deposits of cerium perhydroxide were observed in the intracellular spaces after six days of drying (Figure 2C upper right), indicating a high concentration of H_2_O_2_ accumulation after desiccation. Seeds that were exposed to 100 ppm NO gas showed a reduced number of deposits (Figure 2C middle right), an observation that could be reversed by pretreatment with cPTIO (Figure 2C middle right).

### Exogenous NO application enhanced the recalcitrant seed germination rate

**Figure 3:**
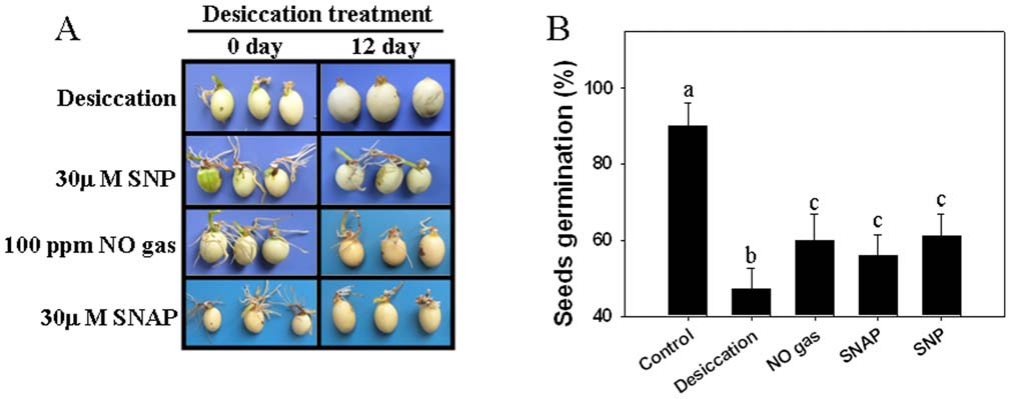
Effects of different NO donors on the *Antiaris toxicaria* seed germination capability. The freshly shed seeds were collected for desiccation plus NO gas exposure. For SNP and SNAP treatment, the fresh seeds were pretreated with 30 µM SNP or SNAP for 12 h, followed by desiccation. After 12 days of desiccation, the seeds were allowed to germinate. The photographs were taken after 10 days of germination (**A**), and the germination percentages (**B**) were also counted after NO donor and desiccation treatment.

Because NO reduces desiccation-induced H_2_O_2_ accumulation and over-accumulation of H_2_O_2_ decreases seed viability, we wanted to know whether NO enhances recalcitrant seed tolerance to desiccation by reducing H_2_O_2_ over-accumulation. Here, in addition to exposure with 100 ppm NO gas, we treated seeds with two other widely used NO donors (sodium nitroprusside (SNP) and S-nitroso-N-acetylpenicillamine (SNAP) prior to desiccation and then observed their germination capabilities. As shown in Figure 3 A&B, NO gas exposure and SNP or SNAP pretreatment enhanced the seed germination rate after desiccation. This result indicates that NO derived from different sources (either direct application or indirect induction) shows a similar positive effect on seed germination and seed desiccation tolerance.

**Figure 4:**
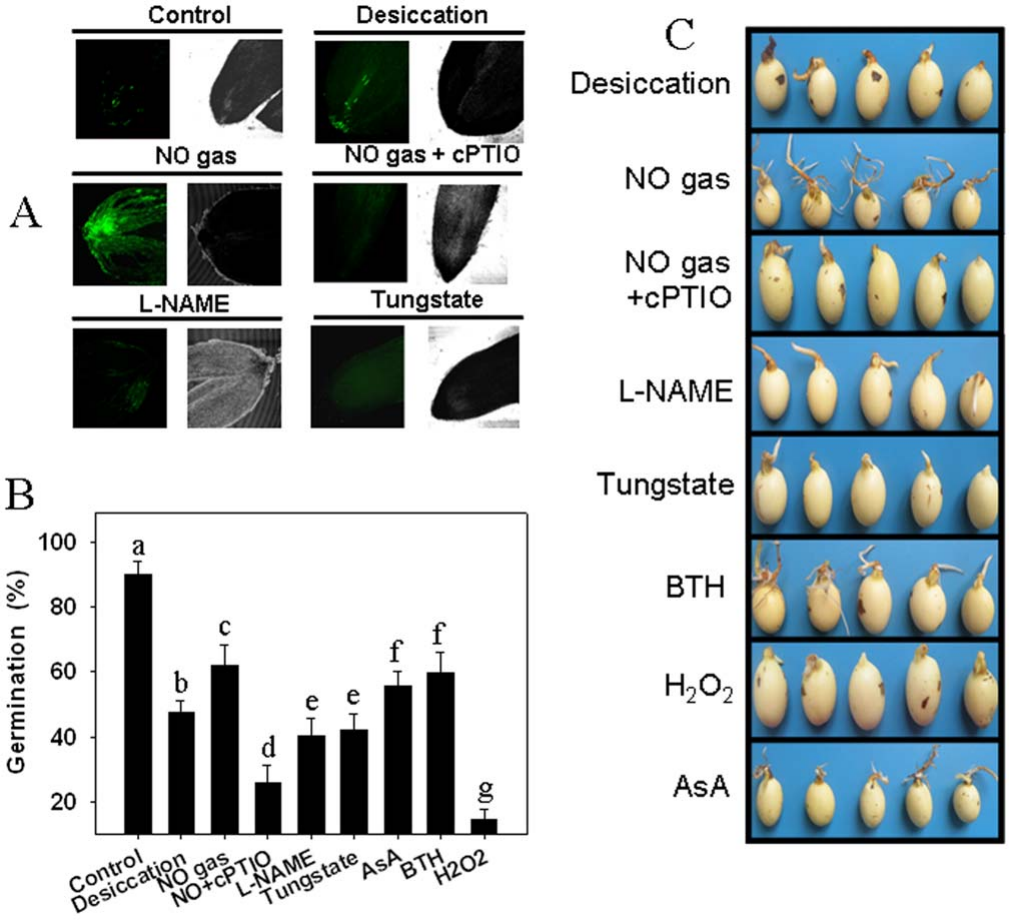
The addition of NO enhanced the recalcitrant *Antiaris toxicaria* seed germination capability after desiccation. **A**) Induction of NO accumulation in seed embryo tips at the early event of desiccation. The freshly shed seeds were pretreated with 10 µM cPTIO, 20 µM L-NAME and 50 µM tungstate for 12 h prior to 2 days of desiccation, and the embryo tips were isolated to detect NO accumulation by fluorescence staining. **B**) **&** **C**) Effects of NO donor, NO scavenger or inhibitor and H_2_O_2_ scavengers on the seed germination capability after 12 days of desiccation treatment. The seeds were pretreated with different chemicals (1 mM AsA, 1 mM BHT, 10 µM cPTIO, 20 µM L-NAME, 50 µM tungstate, 100 nM H_2_O_2_) for 12 h prior to 12 days of desiccation or NO gas exposure. The seed germination percentage (B) was counted. The photographs were taken after 10 days of germination. Data analyzed by one-way ANOVA followed by Tukey's test, different symbols indicate significant differences between treatments (*P*<0.05).

The early and rapid accumulation of NO in the embryo after desiccation was observed by staining with the NO-specific florescence probe 4.5-diaminofluorescein diacetate (DAF-2DA) (Figure 4A). Strong fluorescence was observed in the seed embryos after 12 h of desiccation plus NO gas exposure, and the fluorescence could be efficiently diminished by cPTIO treatment prior to desiccation. L-NAME and tungstate pretreatment also blocked desiccation-induced NO synthesis in the embryo (Figure 4A). We found that L-NAME or tungstate pretreatment further suppressed seed germination when compared to desiccation alone, and the cPTIO pretreatment also reversed the assuaging effect of NO on seed germination after desiccation (Figure 4B&C). Treatment with a reducing agent such as AsA or the antioxidant butylated hydroxy toluene (BTH) prior to desiccation partially improved seed germination capability, while pretreatment with low concentrations of H_2_O_2_ further suppressed seed germination (Figure 4B&C).

### Exogenous NO increases the activity of desiccation-induced antioxidant enzymes

**Figure 5:**
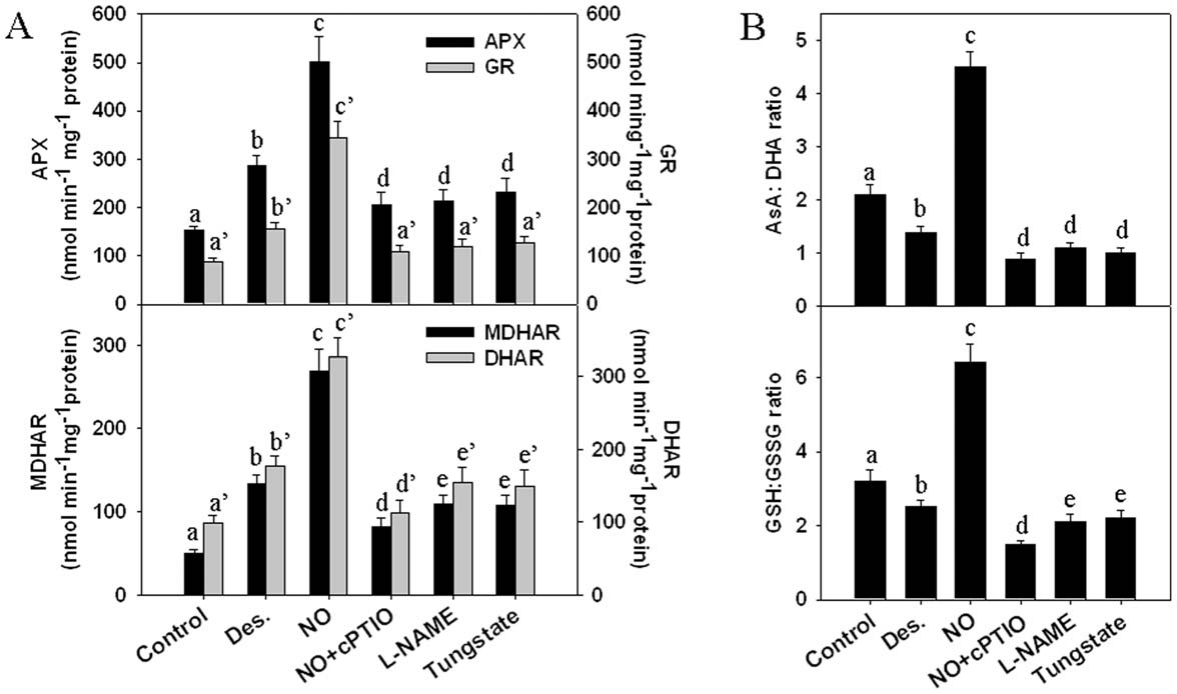
NO activated the metabolism of the ascorbate-glutathione pathway in recalcitrant *Antiaris toxicaria* seeds after desiccation. The freshly collected seeds were pretreated with 10 µM cPTIO, 20 µM L-NAME or 50 µM tungstate for 12 h prior to 12 days of desiccation with or without 100 ppm NO gas exposure. The antioxidant APX, GR, MDHAR, DHAR enzyme activities (**A**) as well as the ASA: DHA and GSH: GSSG ratios (**B**) were measured in the isolated embryos. Data analyzed by one-way ANOVA followed by Tukey's test, different symbols indicate significant differences between treatments (*P*<0.05).

APX, GR, MDHAR and DHAR are the essential antioxidant enzymes involved in the ascorbate-glutathione pathway. Exposing seeds to 100 ppm NO gas increased antioxidant enzyme activities, including APX, GR, MDHAR and DHAR, compared to activities in those seeds treated with desiccation alone (Figure 5A). Pretreatment with the NO scavenger cPTIO and NO metabolism inhibitors L-NAME and tungstate suppressed NO-enhanced antioxidant enzyme activities (Figure 5A). Because the redox status regulates the antioxidant enzyme activities involved in the ascorbate-glutathione pathway and stabilizes the H_2_O_2_ concentration at an appropriate level, we measured the ratios of AsA/DHA and GSH/GSSG in seed embryos after desiccation or different treatments, as shown in Figure 5B. The ratios of GSH/GSSG and AsA/DHA markedly declined after seed desiccation, whereas exposure to NO gas significantly increased these ratios. Suppressing NO accumulation by cPTIO, L-NAME or tungstate reversed NO-induced increases of GSH/GSSG and AsA/DHA ratios during desiccation.

### Regulating the desiccation-induced carbonylation status of antioxidant proteins by nitric oxide in recalcitrant seeds of *A.toxicaria*

**Figure 6:**
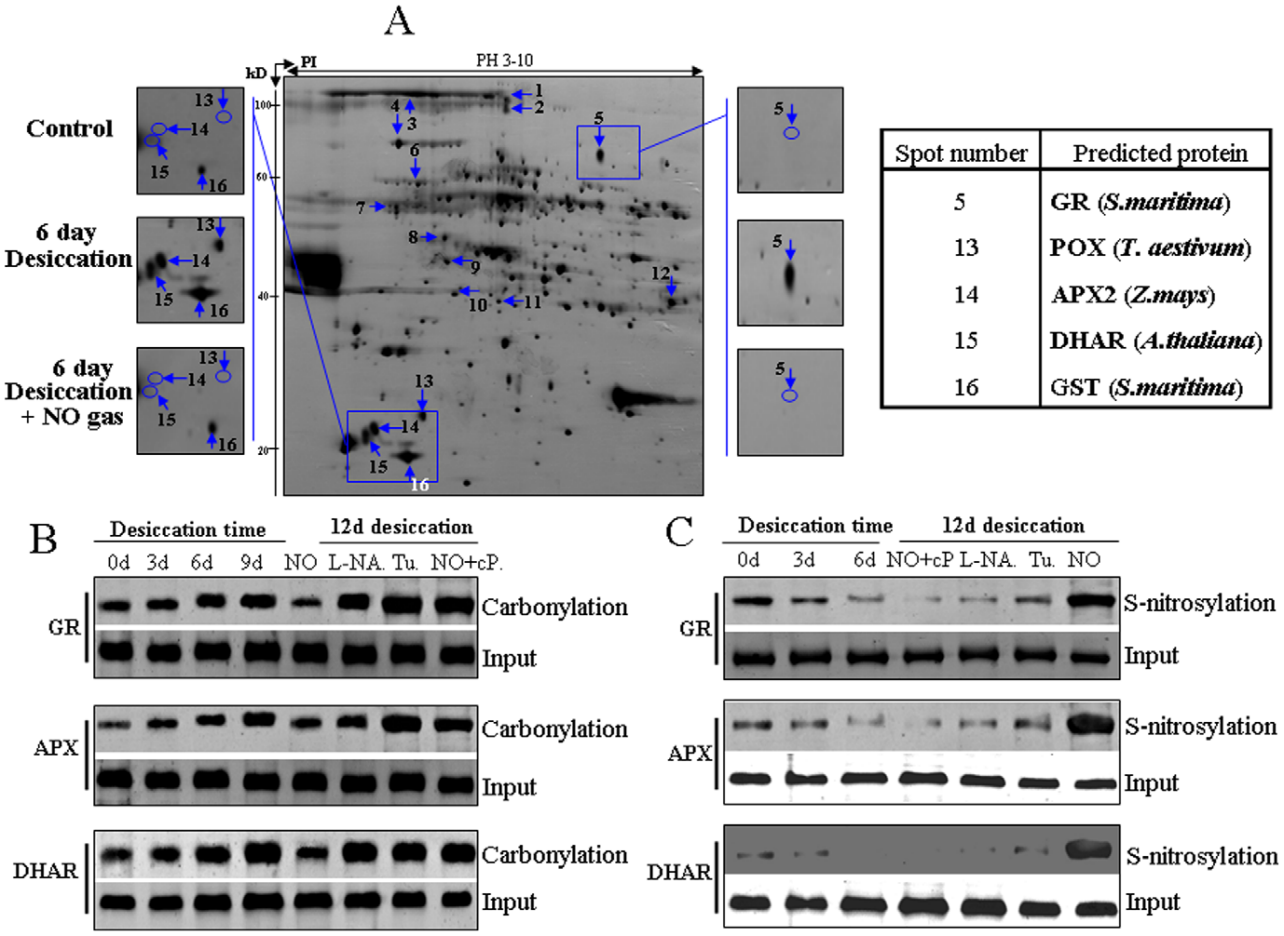
NO and desiccation regulate protein S-nitrosylation and carbonylation status in recalcitrant *Antiaris toxicaria* seeds. **A**) Representative 2D gel of total embryo protein from 12-day-drying seeds. The enlarged windows on the left and right of the gel correspond to gel sections of the control without desiccation, after 12 days of desiccation and after 12 days of desiccation plus NO gas exposure. Spots marked by circles indicate the disappearance of protein spots. Arrows indicate the corresponding proteins identified by MS. The table on the right shows detailed protein spot information. **B**) **&** **C**) Effects of NO gas, NO scavengers and inhibitors on desiccation-induced protein carbonylation (**B**) and S-nitrosylation status (**C**). The shed seeds were desiccated for the indicated time, and the seed embryo proteins were extracted to assay carbonylation and S-nitrosylation status by immunoprecipitation by using the APX, GR and DHAR antibodies. For different inhibitor treatments, the freshly collected seeds were pretreated with 10 µM cPTIO (cP), 20 µM L-NAME (L-NA) or 50 µM tungstate (Tu) for 12 h followed by 12 days of desiccation with or without NO gas exposure.

Oxidative stress in plants is modulated by H_2_O_2_ and NO and leads to oxidative attack on Lys, Arg, Pro or Thr residues of proteins in the form of protein carbonylation. Investigating protein carbonylation profiles will give us more information about the mechanism underlying seed germination during desiccation. We first used 2-D proteomic approach and DNP antibody to detect the entire protein carbonylation status after desiccation or NO treatment. There was a basal level of carbonylated protein in the control embryo protein extracts of *A.toxicaria* (Figure S1). In contrast, the number of carbonylated proteins greatly increased after six days of desiccation treatment, but NO gas exposure during desiccation stress dramatically decreased the extent of desiccation-induced protein carbonylation near the level detected in the control seeds. After digesting and sequencing the isolated protein spots, we identified several carbonylated proteins showing reversible accumulation levels upon treatments with desiccation or NO gas by matching the 2,4-dinitrophenylhydrazine (DNP)-derivative protein spots to Coomassie blue-stained spots (Figure S1). Table S2 presents detailed information about proteins showing different carbonylation statuses after desiccation or NO gas treatment. Among these proteins, the protein spots corresponding to antioxidant enzymes APX, DHAR, GR, GST and PEX showed strong and high carbonylation statuses after desiccation stress, which was reversed by NO gas treatment (Figure 6A). Because these enzymes are involved in the ascorbate-glutathione pathway and are functional in controlling the cellular redox status, their carbonylation status may affect their function; therefore, we further confirmed their carbonylation statuses by using a corresponding antibody and Co-IP. As shown in Figure 6B, we found that desiccation treatment indeed increased carbonylation levels of APX, GR and DHAR enzyme proteins, which were reversed by NO gas treatment. Pretreating the seeds with cPTIO or the NO metabolism inhibitors L-NAME and tungstate restored the antioxidant enzyme carbonylation statuses seen with desiccation treatment alone.

### NO enhanced the S-nitrosylation status of antioxidant enzymes

S-nitrosylation of proteins is another protein modification strategy to alter protein activities. Here, we found that desiccation treatment reduced the S-nitrosylation levels of APX, GR and DHAR proteins (Figure 6C), whereas NO gas exposure increased their S-nitrosylation levels. Treatment with cPTIO, L-NAME or tungstate prior to desiccation reduced the protein S-nitrosylation levels comparing with those of the control or desiccation-treated seed embryo proteins. However, we found that the levels of these antioxidant enzyme proteins themselves were only slightly regulated by desiccation, NO gas exposure or NO scavenger or inhibitor pretreatment compared with their carbonylation or S-nitrosylation levels (Figure S2).

### Increasing protein S-nitrosylation status increased seed tolerance to desiccation

**Figure 7:**
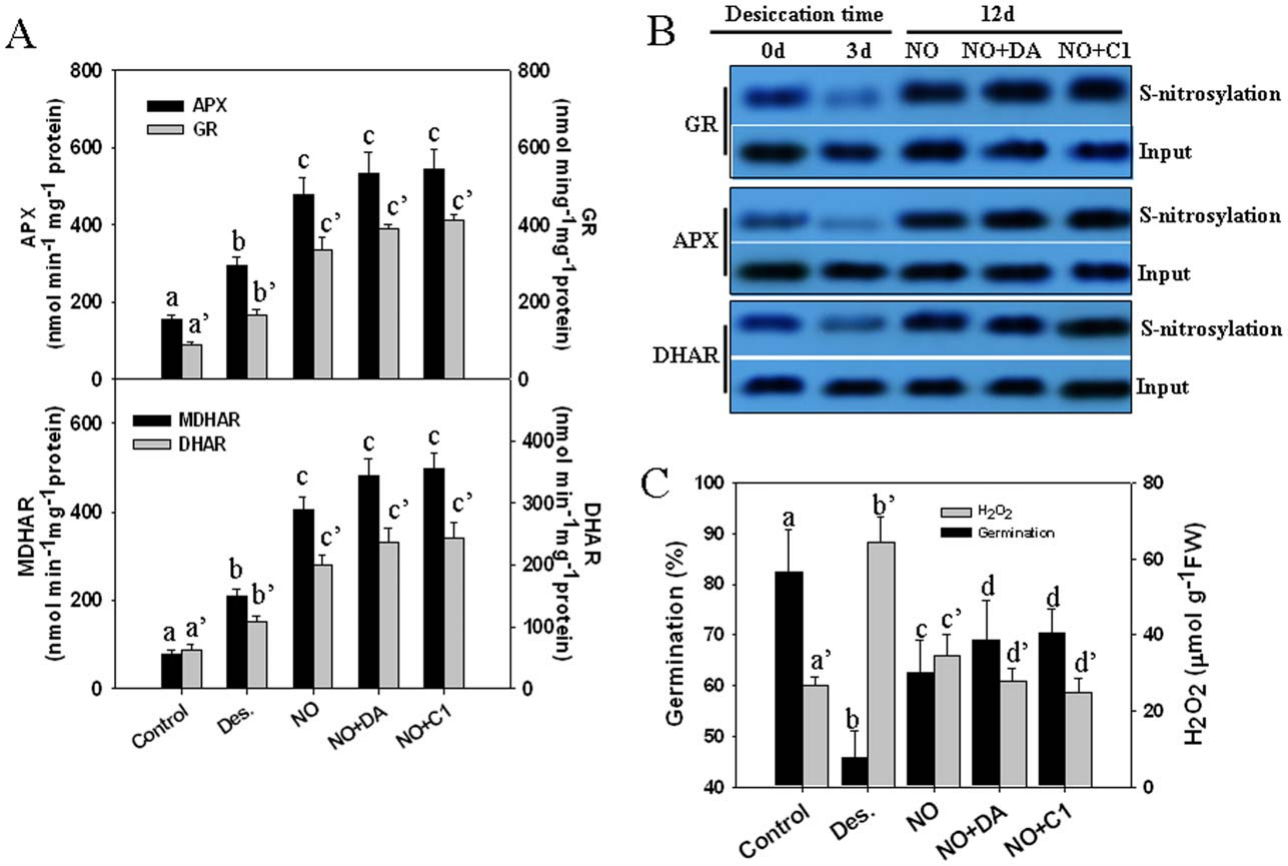
Effects of GNSO inhibitors on APX, GR, MDHAR and DHAR enzyme activities (A), the protein S-nitrosylation status (B) involved in the ascorbate-glutathione pathway and the seed germination capability (C) under desiccation or NO gas treatment. The freshly collected seeds were pretreated with 10 mM DA or 10 µM C1 for 12 h followed by 12 days of desiccation with or without NO gas exposure. The enzyme activities; protein S-nitrosylation status of APX, GR, MDHAR and DHAR; and seed germination were measured. Data analyzed by one-way ANOVA followed by Tukey's test, different symbols indicate significant differences between treatments (*P*<0.05).

S-nitrosoglutathione reductase (GSNOR) is the major enzyme that catalyzes S-nitrosoglutathione (GSNO) metabolism and controls intracellular levels of protein S-nitrosylation [25]. Deleting the reductase gene in yeast and mice abolishes the GSNO-consuming activity and increases the cellular quantity of both GSNO and protein SNO [25]. Dodecanoic acid (DA) and C1 (detailed structures shown in Figure S3) are specific inhibitors of GSNOR enzyme activity [26]. Here, we found that treating the seeds with DA or C1 prior to NO gas exposure promoted the activities of the antioxidant enzymes APX, GR and DHAR (Figure 7A). Pretreatment with both of these GSNOR inhibitors also enhanced the antioxidant protein S-nitrosylation statuses compared with NO gas treatment during desiccation (Figure 7B). Meanwhile, DA or C1 pretreatment enhanced the inhibitory effect of NO on desiccation-induced H_2_O_2_ generation and NO-induced seed tolerance to desiccation, which is reflected in the seed germination rate (Figure 7C).

## Discussion

### NO enhances recalcitrant seed tolerance to desiccation by activating the ascorbate-glutathione pathway and reducing uncontrolled H_2_O_2_ accumulation

A body of evidence has indicated that the ability of seeds to tolerate desiccation and subsequently germinate is related to their ROS levels; Changes in their ascorbate and glutathione levels and the activities of antioxidant enzymes also play a central role during this process [4], [27], [28]. ROS are thought to promote a number of necessary events involved in plant growth, development and adaptation to environment stress [29], [30]; it can also facilitate the breakdown of polysaccharides to loosen the seed coat [31], [32]. ROS can act to deter pathogen infection and spread during seed maturation and germination [33]. However, over-accumulation of ROS as a result of uncontrolled metabolism is one of the most likely reasons for loss of cell membrane integrity and mortality of seeds [34]. In desiccation-tolerant tissues, ROS either have limited accumulation or they are scavenged by antioxidant systems [5], [28]. However, in desiccation-intolerant recalcitrant seeds, ROS cannot be efficiently removed by the immature detoxification system. This is likely the case with other recalcitrant seeds such as sweet chestnut [28]. Here, we have clearly demonstrated that *A. toxicaria* seeds are sensitive to desiccation (Figure 1) and that desiccation is accompanied by a burst of ROS (Figure 2A). Cytochemical localization showed that most desiccation-induced H_2_O_2_ accumulated around the cell wall and intercellular space. The increased reduction of CytC during desiccation would then be expected to lead to higher levels of H_2_O_2_ accumulation and, thus, increased damage to membrane integrity [35]. The addition of exogenous H_2_O_2_ to the *A. toxicaria* seeds during their drying further reduced their post-desiccation viability (Figure 2B), and removing H_2_O_2_ accumulation by AsA or BHT reversed the inhibitory effect of desiccation on seed germination (Figure 2B&C, Figure 4B), demonstrating that ROS is the key factor that renders recalcitrant seeds sensitive to desiccation.

NO is an essential molecule involved in plant seed germination [15], [23], [24]. We observed a rapid accumulation of NO radicals in the embryos of drying seeds using fluorescence staining (Figure 4A). This accumulation could be efficiently suppressed by treatment with the NOS-like enzyme inhibitor L-NAME or the NR enzyme inhibitor tungstate, which indicates that NOS-like enzymes or NR are responsible for desiccation-induced NO synthesis. NO treatment reduced the inhibitory effect of desiccation on seed germination (Figure 3 A&B, Figure 4B&C), and treatment with a NO metabolism scavenger or inhibitor reversed NO-mediated H_2_O_2_ decrease and further reduced the seed germination capability under desiccation. These results reveal that NO efficiently removes desiccation-induced H_2_O_2_ accumulation (Figure 2B&C). The glutathione-ascorbate cycle is responsible for the detoxification of ROS during environmental stress; here, we found that APX, GR, MDHAR and DHAR enzyme activities are induced during the early stages of desiccation and then decline, which causes inefficient removal of ROS. NO gas exposure markedly increases these enzyme activities and enhances reduced ASA and GSH content, which can explain why NO can reduce desiccation-induced ROS accumulation and enhance the seed germination capability.

### NO promoted antioxidant enzyme activities via the regulation of antioxidant protein carbonylation and S-nitrosylation statuses

Protein carbonylation and S-nitrosylation statuses fluctuate depend on environmental stress or stimulation [18], [19], [36]. In Arabidopsis seeds, desiccation strongly increases the extent of protein carbonylation, which might induce seed protein functional properties and enhance their susceptibility to proteolysis [37]. Here, our 2D and western blotting results also demonstrate that desiccation induced carbonylation of several proteins, especially the enzymes involved in the ascorbate-glutathione cycle. This protein carbonylation increase could be reversed by exposure to NO gas, whereas NO gas increased the S-nitrosylation level of the antioxidant enzymes. Both NO-induced effects could be abolished by NO scavenger or inhibitor treatment. These data coincide with previous findings that NO donor treatment increases S-nitrosylation protein levels in *Arabidopsis*. The possible reasons for the NO-induced reversal of desiccation-induced protein carbonylation are that NO reduced the desiccation-induced H_2_O_2_ accumulation or that NO-induced protein S-nitrosylation prevented irreversible protein carbonylation.

Both protein carbonylation and S-nitrosylation are important mechanisms used to regulate protein activities at the post-translational level [38], and they play essential functions in seed physiology; For example, protein carbonylation is involved in H_2_O_2_-induced dormancy alleviation in sunflower seeds [39], and the protein S-nitrosylation process is the switch that regulates the seed germination process in wheat [40]. Recently, Tanou *et al.* [18], [19] suggested that the oxidative carbonylation and S-nitrosylation patterns of leaf proteins are specific molecular signatures for citrus plant vigor under salt stress. In our experiments, we found that GNSOR inhibitor treatment increased the protein S-nitrosylation status, further boosted the NO-activated ascorbate-glutathione cycle, and reversed the desiccation-inhibited seed germination capability. The protein S-nitrosylation status decreases gradually during desiccation, accompanying the decrease in antioxidant enzyme activities as well as seed germination capability. These data suggest that protein carbonylation and S-nitrosylation statuses affect recalcitrant seed sensitivity to desiccation. Additionally, NO-induced protein S-nitrosylation showed a positive effect on activating the ascorbate-glutathione pathway to detoxify ROS damage during desiccation, whereas desiccation-induced ROS caused protein carbonylation to inactivate antioxidant enzyme systems and lead to the subsequent over-accumulation of ROS to damage seed viability. It seems that NO activates enzymes of the ascorbate-glutathione cycle mainly by post-translational protein modulation because desiccation or NO treatment only mildly affected APX, GR and DHAR protein levels.

**Figure 8:**
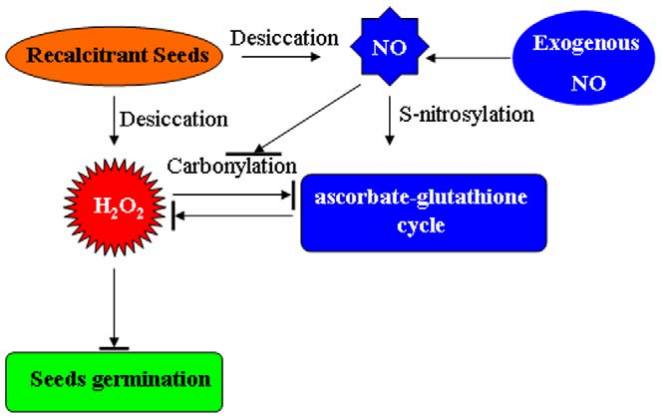
The proposed model to explain the role of NO in recalcitrant seed tolerance to desiccation stress.

In this study, we have demonstrated that NO plays a central role in regulating recalcitrant seed responses to desiccation. Based on our observations, we propose a working model for NO function (Figure 8): unlike orthodox seeds, which have mature antioxidant enzymes to remove the toxic ROS, recalcitrant seeds cannot efficiently scavenge desiccation-induced ROS generation, even though desiccation induced short-lived increases of antioxidant enzyme activity. Desiccation induced small amounts of NO generation, which are inadequate to counteract ROS damage in recalcitrant seeds. Exogenous NO sufficiently induced antioxidant enzyme activities of the ascorbate-glutathione cycle to reduce desiccation-induced ROS accumulation and eventually enhanced recalcitrant seed tolerance to desiccation. It seems that NO is dually functional; first, it activates the ascorbate-glutathione cycle via protein S-nitrosylation, and secondly, it alleviates the inactivation of the ascorbate-glutathione cycle by repressing desiccation-induced protein carbonylation.

## Materials and Methods

### Seed collection and desiccation treatment

Mature fruit of *Antiaris toxicaria* (Pars.) Lesch. (Moraceae) were harvested from trees cultivated at Xishuangbanna Tropical Botanical Garden (21°41′N, 101°25′E; 570-m altitude). Relevant permissions for harvesting mature fruit of *Antiaris toxicaria* (Pars.) Lesch. (Moraceae) is admitted by manager and academic committee of Xishuangbanna Tropical Botanical Garden. The shedding seed pericarps and shells were removed after harvest. The seeds were then rinsed five times and surface-sterilized prior to desiccation in the drying room (4–7°C, 20–25% relative humidity) by air-drying. Seed water content (n = 50×5 replicates) was determined at 110°C for 24 h [4]. For NO gas treatments, seeds were placed in closed desiccators that were linked to a pump allowing the flow of NO (100 ppm NO in a mixture of 15% (v/v) O_2_ and 85% (v/v) N_2_, flow rate 30–50 cm^3^ min^−1^) under the environment described above. For scavenger or inhibitor treatment, the freshly collected seeds were incubated in different concentrations of scavengers or inhibitors for 12 h followed by desiccation or NO gas treatment.

### Seed germination assays

Five replicates of 50 seeds after desiccation or different treatments were placed in the dark on H_2_O-saturated wet filter paper at 25°C. The wet filter papers were replaced every two days. Seeds were judged to have germinated when the radicle pierced the seed coat [15], [23], [24] after two weeks of germination.

### H_2_O_2_ and NO assays

Three replicates of the cotyledons of 10 embryos from the seeds of each treatment group were frozen in liquid N_2_ and ground to a fine powder. The frozen powdered samples were then homogenized in 10 mM Tris-Cl pH 8.0, 1 mM phenylmethylsulfonyl fluoride, 10 mM MgSO_4_, 5 mM KCl, 5 mM NaCl, 10 µM oxyhemoglobin and 10 units ml^−1^ of catalase (100 mg sample ml^−1^ buffer). H_2_O_2_ content was measured according to Hu et al. [9]. For the cytochemical detection of H_2_O_2_, subcellular localization was determined using CeCl_3_ with transmission electron microscopy [41].

NO was measured in vivo in the cotyledons of treated seeds by confocal laser scanning microscopy of DAF-2DA-stained tissues [9]. Epicarp-deprived seeds were thoroughly washed to remove traces of the treatments and were incubated with 10 µM DAF-2DA for 30 min. The embryo cotyledons were removed, and NO-induced fluorescence was observed using a Zeiss confocal laser-scanning microscope (LSM 510 META, Zeiss, Germany) (excitation at 488 nm, emission at 515 nm).

### Enzyme assays

Three replicates of the embryos from 20 seeds from each of the treatments were homogenized in 50 mM sodium phosphate buffer (pH 7.0) containing 1.0 mM ethylenediamine-tetraacetic acid (EDTA), 0.5% (v/v) Triton X-100 and 1% (w/v) polyvinyl-pyrrolidone (PVPP) (100 mg tissue ml^−1^ buffer) (*n.b.* for the analysis of ascorbate peroxidase (APX), monodehydroascorbate reductase (MDHAR) and DHAR, the extraction buffer also contained 5 mM ascorbate). The homogenates were centrifuged (15,000× *g*, 20 min, 4°C), and after determination of their total soluble protein contents by Bradford's assay [42], the supernatants were immediately used for the following standard enzyme assays. Ascorbate peroxidase APX (EC1.11.1.11) activities were determined by measuring the oxidation of ascorbate by following the change in absorbance at A290 nm [43]. Glutathione reductase (GR) (EC1.6.4.2) activities were determined by following the oxidation of NADPH at A340 nm [11]. DHAR (EC1.8.5.1) activities were assayed by measuring the formation of ascorbate at A265 nm [43], and MDHAR (Ec1.6.5.4) activities were assayed by monitoring the change in absorbance at A340 nm [11].

### Ascorbate and Glutathione assays

Following desiccation or different treatments, samples of 50 embryos were homogenized in 5% (w/v) sulfosalicylic acid (for glutathione assay) or 6% TCA (for ascorbate assay) in an ice bath and then centrifuged at 12,000× *g* for 20 min. A 1-mL aliquot of the supernatant was used for further assay. AsA and DHA contents were measured as previous method based on the reduction of Fe^3+^ by AsA in acidic solution [44]. GSH and GSSG contents were measured according to previous method [45].

### Protein extract and two-dimensional gel electrophoresis

Protein extraction and 2D separation was performed according to reproted methods with some modifications [46]. In brief, ten to twenty grams of seeds embryos from different treatment were grounded in liquid nitrogen and total soluble proteins were extracted at 4°C in 5 ml of 50 mM Tris-HCl buffer (pH 7.5) containing 20 mM KCl, 13 mM DTT, 2% (v/v) NP-40, 150 mM PMSF and 1% (w/v) PVP. The homogenates were centrifuged (12,000× *g*, 15 min, 4°C) and the supernatants were added to five volumes of acetone containing 10% (w/v) TCA and 1% (w/v) DTT. The samples were maintained at −20°C for 4 h and then centrifuged (25,000× *g*, 30 min, 4°C). The resulting pellets were washed with acetone containing 1% (w/v) DTT at −20°C for 1 h and then recentrifuged, and the wash was repeated. The final pellets were vaccuum-dried and then dissolved in 8 M urea, 20 mM DTT, 4% (w/v) CHAPS and 2% (w/v) ampholyte (pH 3–10). The samples in ampholyte were vortexed thoroughly for 1 h at room temperature and then centrifuged (25,000× *g*, 20 min, 20°C), and the supernatants were collected for 2D electrophoresis.

Extracted proteins were first separated by isoelectric focusing using gel strips to form an immobilized non-linear pH gradient from 3 to 10 (Immobiline DryStrip pH 3–10 NL, 17 cm; BioRAD, Hercules. CA) and then by SDS–PAGE using 12% polyacrylamide gels. The strips were rehydrated for 16 h in 450 µl of rehydration buffer containing 800 µg of total proteins and a trace of bromophenol blue. The strips were focussed at 20°C for a total of 64 kVh with PROTEAN IEF system (BioRAD, Hercules. CA). After IEF, the strips were equilibrated for 15 min in equilibration buffer (6 M urea, 0.375 M Tris, pH 8.8, 2% (w/v) SDS, 20% (v/v) glycerol and 2% (w/v) DTT). For 2D SDS-PAGE, the strips were placed on top of 12.5% (w/v) SDS-PAGE gels. Gel electrophoresis was performed at 25 mA for 5 h. The gels were stained with colloidal CBB Blue G250^30^. After staining, the gels were scanned using PDQUEST software (Bio-Rad). Parameters were optimized as follows: saliency, 2.0; partial threshold, 4; minimum area, 50. Spots were quantified by determining the ratio of the volume of a single spot to the whole set of spots on the gels. Only changes greater than 2 fold in each of three replicate experiments were used for further analyses.

### Protein identification by mass spectrometry

Individual spots of interest were excised from 2D SDS–PAGE gels using sterile tips and placed in 1.5 ml sterile tubes. Each polyacrylamide piece was rinsed with water, then reduced with 10 mM DTT, alkylated with 55 mM iodoacetamide, and incubated overnight at 37°C with 12.5 ng/µl trypsin (sequencing grade; Roche Diagnostics) in 25 mM NH_4_HCO_3_. The tryptic fragments were extracted, dried, reconstituted with 2% v/v acetonitrile, 0.1% formic acid and sonicated for 10 min. Analysis of tryptic peptides by tandem mass spectrometry was performed on nano liquid chromatography-tandem mass spectrometry analysis using an ultimate 3000 nanoLC(Dionex, Germany). Peptides were loaded in 0.1% formic acid onto a 300 µm ID×5 mm C18 PepMap trap column. Elution and separation of the peptides from the trap column on a 75 µm ID×15 cm C18 pepMap100, 3 µm nanocolumn were done using 0.1% formic acid in acetonitrile at a flow rate of 200 nL/min. To spray sample in the MS, a PicoTip emitter (20 µm ID, 10 µm Tip ID, Woburn, MA, USA) was used with a spray voltage of 1.8 kV. Full scan MS spectra were acquired in the Orbitrap on the 150–2000 *m*/*z* with a resolution of 15 000. The three most intense ions at a threshold above 1000 were selected for collision-induced fragmentation in the linear ion trap at normalized collision energy of 35% after accumulation to a target value of 1000. Dynamic exclusion was employed within 30 s to prevent repetitive selection of the peptides.

Acquired MS/MS spectra were converted to single DTA files using BioWorks (version 3.3.1, Thermo Fisher Scientific). The following parameters were set for creation of the peak lists: parent ions in the mass range with no limitation, one grouping of MS/MS scans and threshold at 100. Precursor ion tolerance was 10.00 ppm. Data were searched using Mascot search engine against all entries in the NCBI database or the DOE database. Carbamidomethylation of cysteines was set as a fixed modification and oxidation of methinine was set as a variable modification. Trypsin was specified as the proteolytic enzyme and one missed cleavage was allowed. The mass tolerance of the precursor ion was set to 10 ppm and that of fragment ions was set to 1 Da. Protein hits were validated if the identification was with at least four top ranking peptides (*P*<0.05). In the case of peptides matching to multiple members of a protein family, the presented protein was selected based on the highest score and the highest member of the matching peptides [47]. The peptide masses and sequences obtained were either matched automatically to proteins in a non-redundant database National Center for Biotechnology Information, NCBI, www.ncbi.nlm.nih.gov using the Mascot MS/MS ions search algorithm (http://www.matrixscience.com) or manual BLAST searches were performed against the current databases. (NCBI, Swiss-Prot, http://expasy.org/sprot/).

### Detection of carbonylated proteins and western blotting

Detection of carbonylated, oxidized proteins was performed by the addition of 2, 4-dinitrophenylhydrazone (DNPH) to protein extract and subsequent immunological detection of the DNP adducts with the monoclonal anti-DNP antibody (Sigma, St. Louis, MO, US) as described previously [18], [19]. In brief, the protein from 2 g of seed embryo after different treatments was extracted at 4°C in 20 mL of extract buffer containing 50 mM Tris-HCl, pH 7.4, 1 mM EDTA, 1% SDS and protease inhibitor cocktail (Complete; Roche Molecular Systems, Alameda, CA, USA). After centrifugation at 12,000× *g* for 10 min, the supernatant was used for carbonylated protein analysis by adding four volumes of 10 mM DNPH (Sigma) and 2 M HCl, and the supernatant was then agitated for 30 min at 4°C. Five volumes of 20/80 ice-cold TCA-acetone containing 1 mM DTT were then added to each sample. The samples were centrifuged for 15 min at 15,000× *g* at 4°C. The precipitated protein was then washed three times with 1 mL of 1∶1 (v/v) ethanol∶ethyl acetate.

For investigating the whole protein carbonylation status by 2D-PAGE analysis, the precipitated protein was resuspended in 100 µL thiourea/urea lysis buffer containing 2% (v/v) Triton X-100 and 20 mM DTT and separated as described above. After 2D-PAGE, the separated proteins were transferred to nitrocellulose sheets (Bio-Rad, Hercules, CA) using standard procedures. Blots were rinsed twice for 5 min in 50 mM Tris-HCl, 150 mM NaCl, pH 7.5 and then incubated for 1 h at 25°C in blocking solution (Roche Diagnostics). After incubation for 1 h with rabbit anti-DNP antibodies (Sigma) in TBS, blots were washed four times in TBS containing 0.05% (v/v) Tween 20 and incubated for 1 h in anti-rabbit secondary antibodies conjugated to horseradish peroxidase (Sigma). Bound antibodies were visualized by using the ECL kit (Sigma). The corresponding carbonylated protein spot were excised, digested and identified as above method.

To investigate the individual protein carbonylation status by 1D-PAGE analysis, the DNPH-treated protein was first immunoprecipitated using specific antibodies to pull down the corresponding protein. In brief, the precipitated protein was resuspended in 0.5 mL incubation buffer containing 50 mM *N*-[Tris (hydroxymethyl)methyl]-2-aminoethane sulfonic acid (TES), 50 mM NaCl, 50 mM KCl, 5 mM MgCl_2_, 1 mM DTT, 10% glycerol, 1 µg ml^−1^ leupeptin, 1 µg ml^−1^ pepstatin A, and 100 µg ml^−1^ PMSF (pH 6.8). The homogenate was centrifuged at 10,000× *g* for 30 min, and the resulting supernatant was further purified by centrifugation at 12,500× *g* for 10 min. For immunoprecipitation, the supernatant is added into 2 µg of purified antibody against APX, GR or DHAR from Agrisera Inc. (Vännäs, Sweden) and incubated at room temperature for 15 min on a rocker table. The polyclonal antibody protein A-coupled Sepharose (P-AS) beads (Sigma) were washed with fresh incubation buffer three times and added into above mixture solution to incubate for 2 h at 4°C. The immunoprecipitate was centrifuged for 3 min at 130× *g*, and the supernatant was discarded. The pellet was washed by resuspension and centrifugation three times with extract buffer. Bound proteins were released from the protein-A-antibody by adding 2× SDS gel loading buffer and boiling for 4 min. Following centrifugation, an equal volume of each sample was fractionated by 1D-PAGE. After transfer of the separated protein onto nitrocellulose sheets (BioRAD), the corresponding carbonylated proteins were detected by western blotting and rabbit anti-DNP antibodies (Sigma) as described above.

### Purification and detection of S-nitrosylated proteins

Purification of S-nitrosylated proteins was performed using the biotin switch assay as described [19]. Briefly, soluble protein was extracted in 10 mL HEN buffer (containing 25 mM HEPES-NaOH, pH 7.7, 1 mM EDTA, 0.1 mM Neocuproine) from 1 g of seed embryo after different treatments in the dark. It was then precipitated by adding four volumes of cooled acetone and centrifuged at 12,000× *g* for 15 min. Next, S-methyl methanethiosulfonate (MMTS, Sigma) was added to a final concentration of 20 mM along with 2% SDS to denature the protein and trap free thiols. Excess MMTS was removed by acetone precipitation, and proteins were solubilized using 0.5 mL HENS (25 mM HEPES-NaOH, pH 7.7, 1 mM EDTA, 0.1 mM neocuproine, 1% SDS). After the addition of 10 mM ascorbate acid and 1 mM biotin-HPDP (N-[6-(biotinamido)-hexyl]-3 (2- pyridyldithio) propionamide, Pierce), the mixture was incubated for 1 h at 22°C in the dark. After acetone precipitation, the protein was solubilized using 200 µL neutralization buffer [20 mM HEPES-NaOH, pH 7.7, 1 mM EDTA, 10 mM NaCl, 0.5% Triton X-100]. Immunoprecipitations were performed as described above using APX, GR or DHAR antibody. The pulled-down proteins were separated by 12% SDS-PAGE and transferred to PVDF membranes (Bio-Rad), the corresponding S-nitrosylated protein band were detected by anti-biotin antibody (Pierce) and the ECL kit (BioRAD). Protein bands corresponding to S-nitrosylated proteins were excised from the gel and protein identification by mass spectrometry were manipulated as described above.

## Supporting Information

Figure S1 **Carbonylated-protein expression signatures of** ***A.toxicaria*** **seed embryo proteins after desiccation or NO gas treatment.** **A**) The control, freshly collected seed embryo proteins without any treatment; **B**) the seed embryo proteins after 12 days of desiccation treatment; **C**) the embryo proteins after 12 days of desiccation plus 100 ppm NO gas exposure; **D**) the reference gel of total soluble protein by Coomassie brilliant blue staining. The identified carbonylated proteins are labeled with arrows.
(TIF)

Figure S2 **The total antioxidant APX, DHAR and GR proteins changer after desiccation or different inhibitors treatment.** The total seeds embryo proteins after 6 day or 12 day of desiccation treatment, or without treatment were extracted for western blot analysis of APX, DHAR and GR protein changes. For inhibitor treatment, the freshly collected seeds were pretreated with 10 µM cPTIO (cP), 20 µM L-NAME (L-NA) or 50 µM tungstate (Tu) for 12 h followed by 12 days of desiccation with or without NO gas exposure, and the antioxidant proteins accumulation levels were analyzed by western blot.
(TIF)

Figure S3 **The structures of the different GSNOR enzyme inhibitors used in this study.**
(DOC)

Table S1 **The correlation (r^2^) between germination capacity and electrolyte leakage, H_2_O_2_ and NO productions in** ***A.toxicaria*** **seeds after 12 days of desiccation treatment.**
(DOC)

Table S2 **Identification of desiccation- or NO-responsive carbonylated proteins in the embryo proteins of desiccated** ***A.toxicaria*** **seeds.**
(XLS)
