# Supplementary figures and images for: Nitric Oxide Enhances Desiccation Tolerance of Recalcitrant Antiaris toxicaria Seeds via Protein S-Nitrosylation and Carbonylation
Source: PLoS One. 2011 Jun 2;6(6):e20714. doi: 10.1371/journal.pone.0020714 (PMC3107241; doi:10.1371/journal.pone.0020714)

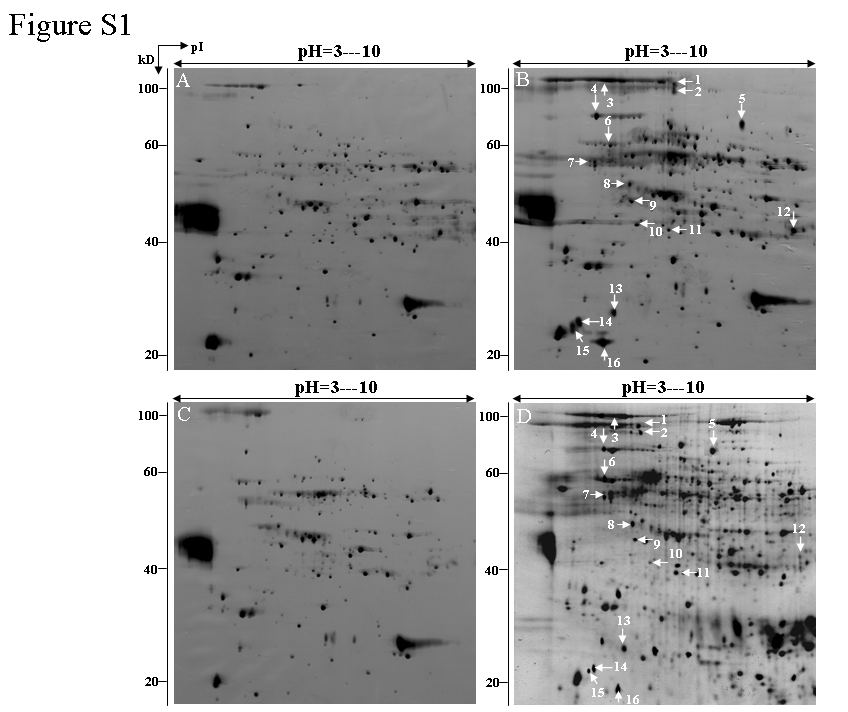

Supplement: Figure S1 — Carbonylated-protein expression signatures of A.toxicaria seed embryo proteins after desiccation or NO gas treatment. A) The control, freshly collected seed embryo proteins without any treatment; B) the seed embryo proteins after 12 days of desiccation treatment; C) the embryo proteins after 12 days of desiccation plus 100 ppm NO gas exposure; D) the reference gel of total soluble protein by Coomassie brilliant blue staining. The identified carbonylated proteins are labeled with arrows. (TIF) [file pone.0020714.s001.tif]

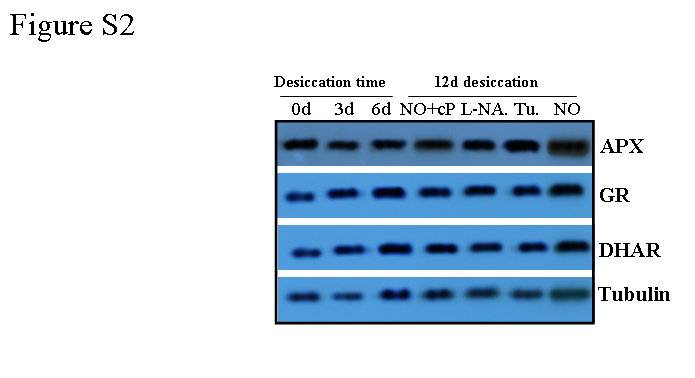

Supplement: Figure S2 — The total antioxidant APX, DHAR and GR proteins changer after desiccation or different inhibitors treatment. The total seeds embryo proteins after 6 day or 12 day of desiccation treatment, or without treatment were extracted for western blot analysis of APX, DHAR and GR protein changes. For inhibitor treatment, the freshly collected seeds were pretreated with 10 µM cPTIO (cP), 20 µM L-NAME (L-NA) or 50 µM tungstate (Tu) for 12 h followed by 12 days of desiccation with or without NO gas exposure, and the antioxidant proteins accumulation levels were analyzed by western blot. (TIF) [file pone.0020714.s002.tif]
