# Supplementary material for: Nitric Oxide Enhances Desiccation Tolerance of Recalcitrant Antiaris toxicaria Seeds via Protein S-Nitrosylation and Carbonylation
Source: PLoS One. 2011 Jun 2;6(6):e20714. doi: 10.1371/journal.pone.0020714 (PMC3107241; doi:10.1371/journal.pone.0020714)
Supplement: Figure S3 — The structures of the different GSNOR enzyme inhibitors used in this study. (DOC) [file pone.0020714.s003.doc]

Figure S3

[
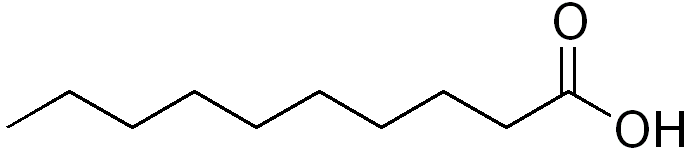
](http://upload.wikimedia.org/wikipedia/commons/0/01/Decanoic_acid.png)

Dodecanoic acid


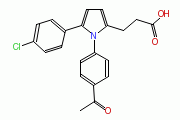


C1: 3-[1-(4-acetylphenyl)-5-phenyl-1H-pyrrol-2-propanoic acid


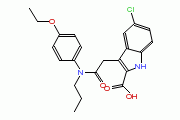


C2: 5-chloro-3-{2-[(4-ethoxyphenyl)(ethyl)amino]-2-oxoethyl}-1H-indole-2-carboxylic acid


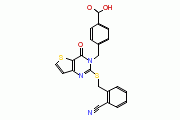


C3: 4-{[2-[(2-cyanobenzyl)thio]-4-oxothieno[3,2-d]pyrimidin-3(4H)-yl]methyl}benzoic acid
