# Supplementary material for: Nitric Oxide Enhances Desiccation Tolerance of Recalcitrant Antiaris toxicaria Seeds via Protein S-Nitrosylation and Carbonylation
Source: PLoS One. 2011 Jun 2;6(6):e20714. doi: 10.1371/journal.pone.0020714 (PMC3107241; doi:10.1371/journal.pone.0020714)
Supplement: Table S1 — The correlation (r2) between germination capacity and electrolyte leakage, H2O2 and NO productions in A.toxicaria seeds after 12 days of desiccation treatment. (DOC) [file pone.0020714.s004.doc]

Table S1

| Germination capacity and electrolyte leakage | Germination capacity and H_2_O_2_ production | Germination capacity and NO production |
| --- | --- | --- |
| 0.9282**  p-0.005 | 0.8770**  p-0.005 | 0.8945**  p-0.005 |
